# Test for the Presence of Mercury

**Published:** 1852-09

**Authors:** 


*Test for the presence of Mercury.*—If a strong solution of iodide of potassium be added to a minute portion of any of the salts of mercury, placed on a sheet of bright copper, a white metallic silvery stain will be developed, which cannot be mistaken, as no other metal presenting a similar appearance is deposited by the same means. In this way corrosive sublimate may be detected, in a solution unaffected by potash or iodide of potassium. In a mixture of calomel and sugar, in the proportion of one of the former to two hundred of the latter, a distinct metallic stain will be detected with one grain of this mixture, containing but 1-200 of its weight of calomel, and the binoxide of mercury can be thus detected, if mixed with 400 times its weight of sugar. This valuable test we owe to Mr. Arthur Morgan.—*Ibid. from Annals of Pharmacy.*

